# Evaluation of three commercial lateral flow immunoassays for the detection of KPC, VIM, NDM, IMP and OXA-48-like carbapenemases

**DOI:** 10.1093/jac/dkae262

**Published:** 2024-08-01

**Authors:** Sara Cuffari, Noemi Aiezza, Alberto Antonelli, Tommaso Giani, Gian Maria Rossolini

**Affiliations:** Department of Experimental and Clinical Medicine, University of Florence, Florence, Italy; Microbiology and Virology Unit, Careggi University Hospital, Florence, Italy; Department of Experimental and Clinical Medicine, University of Florence, Florence, Italy; Microbiology and Virology Unit, Careggi University Hospital, Florence, Italy; Department of Experimental and Clinical Medicine, University of Florence, Florence, Italy; Microbiology and Virology Unit, Careggi University Hospital, Florence, Italy; NARR Joint Laboratory for Antimicrobial Resistance Research and Control, University of Florence-IRCCS Don Gnocchi Foundation, Florence, Italy; Department of Experimental and Clinical Medicine, University of Florence, Florence, Italy; Microbiology and Virology Unit, Careggi University Hospital, Florence, Italy; NARR Joint Laboratory for Antimicrobial Resistance Research and Control, University of Florence-IRCCS Don Gnocchi Foundation, Florence, Italy; Department of Experimental and Clinical Medicine, University of Florence, Florence, Italy; Microbiology and Virology Unit, Careggi University Hospital, Florence, Italy; NARR Joint Laboratory for Antimicrobial Resistance Research and Control, University of Florence-IRCCS Don Gnocchi Foundation, Florence, Italy

The emergence and global dissemination of carbapenemase-producing Gram-negative bacteria (CPGNB) pose a significant public health threat due to the limited therapeutic options for CPGNB infections, which are associated with high mortality rates and a considerable economic and societal burden.^1^

Early identification of CPGNB is useful for timely infection prevention and control measures and therapeutic stewardship, and identification of the resistance mechanisms has become essential following the availability of several new antimicrobial agents with variable coverage depending on resistance mechanism.^2^

Lateral flow immunoassays (LFIAs) are rapid (15–30 min) systems for detection of clinically relevant resistance mechanisms, including carbapenemases. LFIAs require limited technical expertise in the absence of expensive equipment, and are generally less expensive than genotypic methods.^3,4^ Moreover, unlike the latter, LFIA systems detect proteins and are not affected by false positivity in case of silenced genes.^4^ However, false negativity may also occur with LFIA systems in case of low level of production of the target protein (e.g. with weakly expressed MBLs),^5,6^ or of amino acid changes in the epitopes of the target protein recognized by the antibody probe used by the system (e.g. changes at position 179 in the Ω-loop of KPC carbapenemase variants, which confer resistance to avibactam and exhibit impaired carbapenemase activity).^7–11^

In this work we evaluated three commercial LFIA systems for detection of the five most common families of carbapenemases encountered among CPGNB, namely the KPC/IMP/NDM/VIM/OXA-48 Combo Test Kit (KINVO, Medomics Medical Technology, Jiangsu, China), the O.K.N.V.I RESIST-5 (RESIST-5, Coris BioConcept, Gembloux, Belgium) and the NG-Test^®^ DetecTooL CARBA 5 (CARBA-5, NG Biotech, Guipry-Messac, France).

The tested collection comprised 38 previously characterized CPGNB strains of 10 different species, producing several variants of the five carbapenemase families targeted by the LFIA systems, namely KPC (KPC-2, KPC-3, KPC-31, KPC-46, KPC-53, KPC-61, KPC-62, KPC-66, KPC-109, KPC-115, KPC-121, KPC-130), IMP (IMP-2, IMP-8, IMP-12, IMP-16, IMP-13, IMP-19, IMP-30), NDM (NDM-1, NDM-5, NDM-7, NDM-9), VIM (VIM-1, VIM-2, VIM-4) and OXA-48-like (OXA-48, OXA-181, OXA-232). As negative controls, the tested collection also included 10 strains producing other β-lactamases including FIM-1, IMI-2, GES-5, PER-1, FOX-7, NMC-A, OXA-23, OXA-24, OXA-58 and OXA-51, which are not targeted by the LFIA systems (Table 1; and Table S1, available as Supplementary data at *JAC* Online).

**Table 1.** Results with three different lateral-flow immunochromatographic assays for detection of KPC, NDM, VIM, IMP and OXA-48-like carbapenemases in carbapenemase-producing Gram-negative bacteria^a^

Strain	Species	Carbapenemases or other β-lactamases	KINVO system	RESIST-5 system	CARBA 5 system
1Pi	*Klebsiella pneumoniae*	NDM-1	+	++	++
CVB-1	*Escherichia coli*	NDM-1	+	++	++
FI-26246	*Proteus mirabilis*	NDM-1	++	++	++
FI-17181	*K. pneumoniae*	NDM-5	++	++	++
22-1706	*E. coli*	NDM-5	++	++	++
FI-15384	*E. coli*	NDM-7	+	++	++
FI-17144	*K. pneumoniae*	NDM-9	+	++	+
007S(00461882)	*K. pneumoniae*	VIM-1	++	++	++
S61_C01_BS	*Pseudomonas aeruginosa*	VIM-2	++	++	++
VA416/02	*K. pneumoniae*	VIM-4	++	++	++
FI-14/157	*P. aeruginosa*	FIM-1	—	—	—
S137_C02_RS	*P. aeruginosa*	IMP-13	—	++	++
FI-23186	*Enterobacter hormaechei*	IMP-19	++	++	++
AOUC-7/15	*Enterobacter ludwigii*	IMI-2	—	—	—
VA-758/00	*Pseudomonas putida*	IMP-12	++	++	++
Ac54	*Acinetobacter baumannii*	IMP-2	++	++	++
KP0787	*K. pneumoniae*	IMP-8	++	++	++
7723	*P. aeruginosa*	IMP-16	++	++	—
3197	*P. aeruginosa*	IMP-30	++	++	++
FI-24724	*A. baumannii*	OXA-23	—	—	—
VA-566/00	*A. baumannii*	OXA-24	—	—	—
FI-24784	*K. pneumoniae*	OXA-48	++	++	++
ECBZ-1	*E. coli*	OXA-48	++	++	++
123-9962	*A. baumannii*	OXA-51	—	—	—
NV132	*A. baumannii*	OXA-58	—	—	—
FI-9426	*K. pneumoniae*	OXA-232	++	++	++
FI-251	*E. coli*	OXA-232	++	++	++
S49_C01_BS	*P. aeruginosa*	GES-5	—	—	—
S395_C09_RS	*P. aeruginosa*	PER-1	—	—	—
45A02	*K. pneumoniae*	FOX-7	—	—	—
AOUC-8/14	*E. ludwigii*	NMC-A	—	—	—
FI-26814	*Acinetobacter pittii*	NDM-1 + OXA-23	++ (NDM)—(OXA)	++(NDM)—(OXA)	++(NDM)—(OXA)
FI311	*K. pneumoniae*	KPC-3 + VIM-1	++ (KPC) + (VIM)	++	++
Cf-Emp	*Citrobacter freundii*	KPC-2 OXA-181 + VIM-1	++	++	++
FI-17243	*K. pneumoniae*	NDM-5 + OXA-181	++ (NDM), ++ (OXA)	++	++
FI150	*K. pneumoniae*	NDM-5 + OXA-232	++ (OXA-48-like), +(NDM)	++	++
001	*P. mirabilis*	KPC-2	++	++	—
004	*P. mirabilis*	KPC-2	++	++	++
KP-PG-0618	*K. pneumoniae*	KPC-31^b^ (D179Y compared with KPC-3, in Ω-loop)	+	—	—
FI-20012	*K. pneumoniae*	KPC-46^b^ (L168P compared with KPC-3, in Ω-loop)	++	++	++
FI-13547	*K. pneumoniae*	KPC-53^b^ (L167E168 duplication compared with KPC-3, in Ω-loop)	++	++	+
FI-19259	*K. pneumoniae*	KPC-61^b^ (S171P compared with KPC-3, in Ω-loop)	+	+	+
FI-18706	*K. pneumoniae*	KPC-62^b^ (L168Q compared with KPC-3, in Ω-loop)	++	+	++
FI-26529	*K. pneumoniae*	KPC-66^b^ (E167_L168del compared with KPC-3, in Ω-loop)	++	++	++
FI-21194	*K. pneumoniae*	KPC-109^b^ (ins_270_KYNKDD compared with KPC-3, in 270-loop)	+	++	++
FI-26754	*K. pneumoniae*	KPC-115^b^ (L168P, D169_S170del compared with KPC-3, in Ω-loop)	++	++	+
FI-15565	*K. pneumoniae*	KPC-121^b^ (S181 duplication compared with KPC-3, in Ω-loop)	+	—	—
FI-13908	*K. pneumoniae*	KPC-130^b^ (D179G compared with KPC-3, in Ω-loop)	++	+	—^c^
**Total**			37/38 (97.4%)	36/38 (94.7%)	33/38 (86.8%)

^a^++ indicates a strong signal; +indicates a weak signal; — indicates a negative signal.

^b^Amino acid alterations associated with resistance to ceftazidime/avibactam and, in some cases also with reduced carbapenemase activity.^9^

^c^The test was weakly positive (+) after repetition with a higher inoculum compared with recommended instructions.

All tested strains were grown on CHROMID CPSE medium (bioMèrieux, Marcy l’Etoile, France) for 16–24 h at 35 ± 1°C. For testing, a 1 µL loopful of each strain was resuspended in the extraction buffers provided by the manufacturers, and the suspension was added to the sample well of the test cassette following the manufacturers’ instructions. The results were visually read within 15 min. In case of unexpected negativity, the test was repeated from the same colony (to exclude the presence of heterogeneous populations), using a standard and a higher (approximately 2-fold) bacterial inoculum.

All three systems exhibited 100% specificity, and detected all tested variants of the OXA-48-like, NDM and VIM enzymes (Table 1). Regarding IMP carbapenemases, most tested variants were detected by all systems, but IMP-13 was not detected by KINVO, and IMP-16 was not detected by CARBA-5, even when using higher inocula (Table 1). Regarding KPC enzymes, most tested variants were detected by all systems, but KPC-31 and KPC-121 were not detected by RESIST-5 and CARBA-5, even when using higher inocula, and KPC-130 was not detected by CARBA-5 (unless using a higher inoculum). Moreover, one of the two KPC-2–producing *Proteus mirabilis* tested negative with CARBA-5, even when using a higher inoculum, likely due to lower carbapenemase production by this strain (Table 1). Finally, coproduction of enzymes of different families (e.g. KPC-3 + VIM-1, KPC-2 + VIM-1 + OXA-181, NDM-5 + OXA-181, NDM-5 + OXA-232, NDM-1 + OXA-23) was always correctly detected (Table 1).

LFIA systems for carbapenemase detection in CPGNB are increasingly popular in laboratory practice for their rapidity and ease of use. However, the emergence of carbapenemase variants is posing some challenges to their diagnostic sensitivity.^7–13^ This problem could increase with the rising selective pressure generated by the use of novel β-lactam/β-lactamase inhibitor combinations, which might promote further diversification of allelic variants.

To the best of our knowledge, this is the first evaluation of the KINVO system, which was able to detect some Ω-loop variants of KPC (e.g. KPC-31, KPC-121) that are missed by the other LFIA systems, but missed in turn the IMP-13 variant.

A limitation of this study was that only some variants of the major carbapenemase families were tested. Testing of additional variants (including also less common variants, such as OXA-48-like presenting a deletion in β5-β6 loop) will be needed to further expand knowledge about the performance of these diagnostic systems.

## Supplementary Material

dkae262_Supplementary_Data

## Supplementary data

Table S1 is available as Supplementary data at *JAC* Online.
