# Supplementary material for: Evaluation of three commercial lateral flow immunoassays for the detection of KPC, VIM, NDM, IMP and OXA-48-like carbapenemases
Source: J Antimicrob Chemother. 2024 Aug 1;79(10):2724–7. doi: 10.1093/jac/dkae262 (PMC11441996; doi:10.1093/jac/dkae262)
Supplement: dkae262_Supplementary_Data [file dkae262_supplementary_data.pdf]

Supplementary Table 1. Strains list.

Strain name	Species	Strain Reference
KP-PG-0618	*K. pneumoniae*	1
FI-20012	*K. pneumoniae*	This study
FI-13547	*K. pneumoniae*	2
FI-19259	*K. pneumoniae*	This study
FI-18706	*K. pneumoniae*	This study
FI-26529	*K. pneumoniae*	This study
FI-21194	*K. pneumoniae*	3
FI-26754	*K. pneumoniae*	This study
FI-15565	*K. pneumoniae*	This study
FI-13908	*K. pneumoniae*	This study
001	*P. mirabilis*	20
004	*P. mirabilis*	This study
1Pi	*K. pneumoniae*	4
CVB-1	*E. coli*	5
FI-26246	*P. mirabilis*	This study
FI-17181	*K. pneumoniae*	This study
22-1706	*E. coli*	6
FI-15384	*E. coli*	This study
FI-17144	*K. pneumoniae*	7
007S(00461882)	*K. pneumoniae*	8
S61_C01_BS	*P. aeruginosa*	9
VA416/02	*K. pneumoniae*	10
FI-14/157	*P. aeruginosa*	11
Ac54	*A. baumannii*	21
KP0787	*K. pneumoniae*	22
VA-758/00	*P. putida*	23
S137_C02_RS	*P. aeruginosa*	12
7723	*P. aeruginosa*	25
FI-23186	*E. hormaechei*	This study
3197	*P. aeruginosa*	24
AOUC-7/15	*E. ludwigii*	6
FI-24724	*A. baumannii*	This study
VA-566/00	*A. baumannii*	13
FI-24784	*K. pneumoniae*	This study
ECBZ-1	*E. coli*	14
123-9962	*A. baumannii*	15
NV132	*A. baumannii*	15

FI-9426	*K. pneumoniae*	16
FI-251	*E. coli*	16
S49_C01_BS	*P. aeruginosa*	9
S395_C09_RS	*P. aeruginosa*	9
45A02	*K. pneumoniae*	17
AOUC-8/14	*E. ludwigii*	18
FI-26814	*A. pittii*	This study
FI311	*K. pneumoniae*	This study
Cf-Emp	*C. freundii*	19
FI-17243	*K. pneumoniae*	This study
FI150	*K. pneumoniae*	This study

Supplementary References

1. Antonelli A, Giani T, Di Pilato V, *et al.* KPC-31 expressed in a ceftazidime/avibactam-resistant *Klebsiella pneumoniae* is associated with relevant detection issues. *J Antimicrob Chemother* 2019; **74**: 2464–6.
2. Di Pilato V, Aiezza N, Viaggi V, *et al.* KPC-53, a KPC-3 Variant of clinical origin associated with reduced susceptibility to ceftazidime-avibactam. *Antimicrob Agents Chemother* 2020; **65**: e01429-20.
3. Di Pilato V, Codda G, Niccolai C, *et al.* Functional features of KPC-109, a novel 270-loop KPC-3 mutant mediating resistance to avibactam-based β-lactamase inhibitor combinations and cefiderocol. *Int J Antimicrob Agents* 2024; **63**: 107030.
4. Di Pilato V, Henrici De Angelis L, Aiezza N, *et al.* Resistome and virulome accretion in an NDM-1-producing ST147 sublineage of *Klebsiella pneumoniae* associated with an outbreak in Tuscany, Italy: a genotypic and phenotypic characterisation. *The Lancet Microbe* 2022; **3**: e224–34.
5. D'Andrea MM, Venturelli C, Giani T, *et al.* Persistent carriage and infection by multidrug-resistant *Escherichia coli* ST405 Producing NDM-1 carbapenemase: report on the first italian cases. *J Clin Microbiol* 2011; **49**: 2755–8.
6. Coppi M, Antonelli A, Giani T, *et al.* Multicenter evaluation of the RAPIDEC® CARBA NP test for rapid screening of carbapenemase-producing *Enterobacteriaceae* and Gram-negative nonfermenters from clinical specimens. *Diagn Microbiol Infect Dis* 2017; **88**: 207–13.
7. Le Terrier C, Nordmann P, Buchs C, *et al.* Wide dissemination of Gram-negative bacteria producing the taniborbactam-resistant NDM-9 variant: a One Health concern. *J Antimicrob Chemother* 2023; **78**: 2382–4.

8. David S, Cohen V, Reuter S, *et al.* Integrated chromosomal and plasmid sequence analyses reveal diverse modes of carbapenemase gene spread among *Klebsiella pneumoniae*. *Proc Natl Acad Sci* 2020; **117**: 25043–54.
9. Giani T, Arena F, Pollini S, *et al.* Italian nationwide survey on *Pseudomonas aeruginosa* from invasive infections: activity of ceftolozane/tazobactam and comparators, and molecular epidemiology of carbapenemase producers. *J Antimicrob Chemother* 2018; **73**: 664–71.
10. Luzzaro F, Docquier J-D, Colinon C, *et al.* Emergence in *Klebsiella pneumoniae* and *Enterobacter cloacae* Clinical Isolates of the VIM-4 Metallo-β-Lactamase Encoded by a Conjugative Plasmid. *Antimicrob Agents Chemother* 2004; **48**: 648–50.
11. Pollini S, Maradei S, Pecile P, *et al.* FIM-1, a New Acquired Metallo-β-Lactamase from a *Pseudomonas aeruginosa* Clinical Isolate from Italy. *Antimicrob Agents Chemother* 2013; **57**: 410–6.
12. Giani T, Arena F, Pollini S, *et al.* Italian nationwide survey on *Pseudomonas aeruginosa* from invasive infections: activity of ceftolozane/tazobactam and comparators, and molecular epidemiology of carbapenemase producers. *J Antimicrob Chemother* 2018; **73**: 664–71.
13. D'Andrea MM, Giani T, D'Arezzo S, *et al.* Characterization of pABVA01, a plasmid encoding the OXA-24 carbapenemase from italian isolates of *Acinetobacter baumannii*. *Antimicrob Agents Chemother* 2009; **53**: 3528–33.
14. Giani T, Conte V, Di Pilato V, *et al. Escherichia coli* from Italy producing OXA-48 carbapenemase encoded by a novel Tn 1999 transposon derivative. *Antimicrob Agents Chemother* 2012; **56**: 2211–3.
15. Principe L, Piazza A, Giani T, *et al.* Epidemic diffusion of OXA-23-Producing *Acinetobacter baumannii* isolates in Italy: results of the first cross-sectional countrywide survey gilligan PH, ed. *J Clin Microbiol* 2014; **52**: 3004–10.
16. Riccobono E, Cervini C, Morecchiato F, *et al*. Evaluation of Revogene® Carba C assay for the rapid detection of carbapenemase genes in gram-negative pathogens. *Diagn Microbiol Infect Dis* 2022; **102**: 115614.
17. Di Pilato V, Arena F, Giani T, *et al*. Characterization of pFOX-7a, a conjugative IncL/M plasmid encoding the FOX-7 AmpC-type β-lactamase, involved in a large outbreak in a neonatal intensive care unit. *J Antimicrob Chemother* 2014; **69**: 2620–4.
18. Antonelli A, D'Andrea MM, Di Pilato V, *et al*. Characterization of a novel putative Xer-dependent integrative mobile element carrying the $\mathit{bla}_{\mathrm{NMC\text{-}A}}$ carbapenemase gene, inserted into the chromosome of members of the *Enterobacter cloacae complex*. *Antimicrob Agents Chemother* 2015; **59**: 6620–4.
19. Riccobono E, Salvetti S, Coppi M, *et al*. *Citrobacter freundii* resistant to novel β-lactamase inhibitor combinations and cefiderocol, co-producing class A, B and D carbapenemases encoded by transferable plasmids. *J Antimicrob Chemother* 2023; **78**: 1677–82.

20. Di Pilato V, Chiarelli A, *et al*. Complete Genome Sequence of the First KPC-Type Carbapenemase-Positive Proteus mirabilis Strain from a Bloodstream Infection. *Genome Announc* 2016; **4**: e00607-16.
21. Riccio ML, Franceschini N, Boschi L, *et al*. Characterization of the metallo-beta-lactamase determinant of *Acinetobacter baumannii* AC-54/97 reveals the existence of $bla_{(IMP)}$ allelic variants carried by gene cassettes of different phylogeny. *Antimicrob Agents Chemother* 2000; **44**:1229-35.
22. Yan JJ, Ko WC, Wu JJ. Identification of a plasmid encoding SHV-12, TEM-1, and a variant of IMP-2 metallo-beta-lactamase, IMP-8, from a clinical isolate of *Klebsiella pneumoniae*. *Antimicrob Agents Chemother* 2001; **45**: 2368-71.
23. Docquier JD, Riccio ML, Mugnaioli C, *et al*. IMP-12, a new plasmid-encoded metallo-beta-lactamase from a *Pseudomonas putida* clinical isolate. *Antimicrob Agents Chemother* 2003; **47**: 1522-8.
24. Edelstein MV, Skleenova EN, Shevchenko OV, *et al*. Spread of extensively resistant VIM-2-positive ST235 *Pseudomonas aeruginosa* in Belarus, Kazakhstan, and Russia: a longitudinal epidemiological and clinical study. *Lancet Infect Dis* 2013; **13**: 867-76.
